# Physical Health Screening in a Mental Health Setting

**DOI:** 10.1192/bjo.2022.88

**Published:** 2022-06-20

**Authors:** Laura Middleton, Katherine Ashcroft, Alex Mather, Georgina Gargan, Abby Older, Andrew Mitchell, Elizabeth Shaw

**Affiliations:** 1 Health Education England North West School of Psychiatry, Liverpool, United Kingdom; 2 Countess of Chester Hospital NHS Foundation Trust, Chester, United Kingdom; 3 Cheshire and Wirral Partnership NHS Foundation Trust, Chester, United Kingdom

## Abstract

**Aims:**

Research demonstrates greater mortality and physical health morbidity in those with mental illness, as compared to the general population. National Health Service (NHS) England has introduced policies to reflect this and promote improvements in physical healthcare for mental health patients. Inpatient admission provides a valuable opportunity to action such recommendations and offer a detailed health review, guided by local frameworks. A new annual audit commenced in Cheshire and Wirral Partnership NHS Foundation Trust (CWP) assesses admission physical health screening on its adult acute inpatient wards.

**Methods:**

Audit standard was 100% compliance to CWP's admission pathway (Policy CP35). Parameters included doctor's review, medical history, physical examination, drug history, medication chart, allergy status, venous thromboembolic risk, blood tests, electrocardiogram (ECG), physiological observations, smoking history, body mass index (BMI) and falls risk. Data were collected retrospectively for all patients admitted or transferred to Juniper Ward, an acute adult inpatient unit in Bowmere Hospital in Chester, during October 2020 (cycle 1) and September 2021 (cycle 2). Different months were assessed due to senior staff changes in October 2021.

**Results:**

30 patients were identified in 2020 and 37 in 2021. In 2020 the most consistently achieved parameters were, in order, medication chart/drug history, doctor's review and past medical history. In 2021 the most consistently achieved parameters were medication chart/drug history, smoking status and past medical history. Across both years completion of the cardiometabolic tool was lowest, although this improved from 6.7% to 16.2%. In 2020 there were 5 parameters achieving <50% compliance (cardiometabolic, physiological observations, smoking status, BMI and falls risk). In 2021 this reduced to 3 parameters (doctor's review, cardiometabolic tool, falls risk). Local policy was updated following the 2020 results, amending the criteria for doctor's review from *commenced* within 6 hours, to *completed* within 12 hours. Improvement was seen in all other areas in 2021, with medication chart/drug history documentation achieved in 100% of admissions.

**Conclusion:**

Generalised improvement was seen following the 2020 audit, although only one parameter reached 100% compliance and most remained under 75%. The first cycle led to a policy change with respect to the doctor's review timeframe, although this limited direct comparison between years. A flow chart will be trialled on Juniper Ward, highlighting required tasks and assigning ownership to specific team members. The local Medical Education team were also made aware of the results to inform junior doctor induction. The audit will be repeated in Autumn 2022.

